# New Insights into the State Trapping of UV-Excited Thymine

**DOI:** 10.3390/molecules21111603

**Published:** 2016-11-23

**Authors:** Ljiljana Stojanović, Shuming Bai, Jayashree Nagesh, Artur F. Izmaylov, Rachel Crespo-Otero, Hans Lischka, Mario Barbatti

**Affiliations:** 1 Aix Marseille Univ., CNRS, ICR, Marseille, France; ljiljana.stojanovic@univ-amu.fr (L.S.); shuming.bai@univ-amu.fr (S.B.); 2 Chemical Physics Theory Group, Department of Chemistry, University of Toronto, Toronto, ON M5S 3H6, Canada; jnagesh@chem.utoronto.ca (J.N.); artur.izmaylov@utoronto.ca (A.F.I.); 3 Department of Physical and Environmental Sciences, University of Toronto Scarborough, Toronto, ON M1C 1A4, Canada; 4 School of Biological and Chemical Sciences, Queen Mary University of London, Mile End Road, London E1 4NS, UK; r.crespo-otero@qmul.ac.uk; 5 School of Pharmaceutical Sciences and Technology, Tianjin University, Tianjin 300072, China; hans.lischka@univie.ac.at; 6 Department of Chemistry and Biochemistry, Texas Tech University, Lubbock, TX 79409, USA

**Keywords:** computational theoretical chemistry, photochemistry, nonadiabatic dynamics, ultrafast processes, surface hopping, nucleobases, thymine

## Abstract

After UV excitation, gas phase thymine returns to a ground state in 5 to 7 ps, showing multiple time constants. There is no consensus on the assignment of these processes, with a dispute between models claiming that thymine is trapped either in the first (S_1_) or in the second (S_2_) excited states. In the present study, a nonadiabatic dynamics simulation of thymine is performed on the basis of ADC(2) surfaces, to understand the role of dynamic electron correlation on the deactivation pathways. The results show that trapping in S_2_ is strongly reduced in comparison to previous simulations considering only non-dynamic electron correlation on CASSCF surfaces. The reason for the difference is traced back to the energetic cost for formation of a CO π bond in S_2_.

## 1. Introduction

After UV excitation, gas phase thymine returns to the ground state within 5 to 7 ps [1]. In the 14 years since ultrafast time-resolved spectroscopy of this molecule was reported for the first time [2], this seems to be the only consensus on the interpretation of its photophysics. The elusive nature of thymine’s photophysics stems from the difficulty of assigning multiple time constants underlying its time-resolved photoelectron spectrum [1,2,3,4,5,6,7,8,9,10]. In fact, a literature survey (see Table 1) reveals that there is no full agreement on even how many time constants are implicit in those spectra [1,3,7]. Most of results tend to converge to a three time-constants scheme, with a short sub-picosecond time constant of about 100–200 fs, a picosecond time constant of about 6 ps, and a nanosecond time constant reaching nearly 300 ns.

Taking the picosecond time constant as an indication of internal conversion to the ground state—which is the most common interpretation—leaves thymine with the longest excited state lifetime among the isolated nucleobases [7,11]. This fact is in itself puzzling, as thymine’s potential energy surfaces obtained from high-level computational simulations are very similar to those of other short-lived pyrimidines (uracil, for instance), to justify the time constant differences [12].

Computational simulations have revealed that thymine internal conversion after UV excitation should involve two singlet excited adiabatic states, S_1_ and S_2_ [12,13]. These states may have nπ* or diverse ππ* characters along the reaction paths. There is an extended accessible crossing seam region between S_2_ and S_1_ (ππ*/nπ*) [14], as well as between S_1_ and the ground state (ππ*/S_0_ and nπ*/S_0_) [15]. A long-lived triplet ππ* state plays a role over longer scales [1,9,16,17] not explored here.

In earlier works, thymine’s shortest time constant has been assigned to direct internal conversion to ground state along a ππ* pathway. Such a model—we will refer to it as the “fast ππ* model”—was proposed on the basis of either analyses of ab initio potential energy surfaces [18,19] or surface hopping dynamics on semi-empirical surfaces [20]. Nevertheless, the agreement between these works is restricted to this sub-picosecond step: while ref. [18] proposes that the picosecond step would occur due to a delayed ππ* deactivation, ref. [19] attributes this longer step to a sequential ππ* → nπ* → S_0_ conversion. Ref. [20], on its turn, also predicts a sequential ππ* → nπ* → S_0_ conversion process, but occurring in the sub-picosecond scale.

A different photophysical model was proposed in ref. [13] and later corroborated by ref. [15], both on the basis of analysis of ab initio potential energy surfaces. This model—the “S_1_ trapping model”—assigns the short time constant to a fast S_2_ (ππ*) → S_1_ (nπ*) transition, while the picosecond time constant is assigned to a S_1_ (nπ*) → S_0_ transition. Thus, according to this interpretation, the elongated picosecond time constant of thymine would be caused by a trapping in the S_1_ state.

The S_1_ trapping model has been popular among experimentalists, as it apparently correlates well with the electron binding energy (*E_b_*) observed in time-resolved experiments [1,6,21]. Their argument goes as follows: the first ionization potential (IP) of thymine is a π hole, while the second is an n hole. Thus, spectral signals at low *E_b_* near the first IP should be caused by probing the ππ* state, while spectral signals at large *E_b_* near the second IP should be caused by probing the nπ* state. Because the signal in the picosecond scale comes from large *E_b_*, this would be an evidence that thymine is in the nπ* state during the picosecond regime. The problem with this argument is that it assumes that electrons are usually ejected with the maximum electron kinetic energy (or minimum *E_b_*, near the IP). This is correct only for ionization of stationary states. When probing wave packets, a much wider range of electron kinetic energies should be expected [22]. Thus, while it is true that spectral signal near the first IP should be essentially due to ππ* probing, the signal near the second IP contains not only information from nπ* probing, but also information from ππ* probing of electrons being ejected with low kinetic energy.

Although this analysis of the electron kinetic energy does not disprove the S_1_ trapping model (which is good for us, as will be advocating for it later), it at least reduces its strength. If that were not enough, there is still a third model for thymine deactivation in direct competition with it, the “S_2_ trapping model”.

S_2_ trapping was first proposed on the basis of multiple spawning dynamics on complete active space self-consistent field (CASSCF) surfaces [23]. These simulations, limited to a short sub-picosecond time scale, showed that after excitation into S_2_ (ππ*) state, conversion to S_1_ (nπ*) was unexpectedly slow. This led to the hypothesis that the picosecond time constant was due to thymine’s trapping in S_2_, while the short sub-picosecond time constant was caused by relaxation of the ππ* state between the Franck-Condon region and the S_2_ minimum.

The S_2_ trapping model got some additional support from surface hopping dynamics still on CASSCF surfaces [14,24]. These simulations were performed on longer time scales than in the original multiple spawning simulations and confirmed that slow S_2_ → S_1_ transfer. However, the surface hopping results also added a new layer of complexity, as they showed that the S_2_ trapping could only explain a delay of about 2 ps in the lifetime; therefore, to reach a 6 ps time constant, thymine should also be trapped in S_1_ after the S_2_ → S_1_ transition. A final bit of complexity was later added to the model by wave packet dynamics [25]. It showed that even the common hypothesis that only the ππ* state is excited needs to be relaxed, as vibronic couplings could lead to a substantial nπ* population within the first 50 fs of dynamics, with the remaining ππ* population trapped in a flat S_2_. Thus, together, these results from surface hopping and wave packet dynamics seemed to point out to a new “S_2_ and S_1_ trapping model”.

A couple of years ago, however, the S_2_ trapping hypothesis was challenged by time-resolved Auger spectroscopy [4], which combined with spectrum simulations at CIS level made a good case towards a population transfer to the nπ* state within 200–300 fs. Once more, the S_1_ trapping model would be invoked to explain the picosecond time constant.

Giving this cloudy state of affairs, we decided to revisit thymine dynamics. Although multiple spawning and surface hopping dynamics have provided some compelling arguments for the S_2_ trapping, these simulations have a common major weak point: they were based on CASSCF surfaces. CASSCF does an excellent job recovering non-dynamic electron correlation near intersections between the ground and the first excited states, but it neglects most dynamic electron correlation, which is present through the whole reaction path. This poses a serious problem: the key step to determine the occurrence (or not) of the S_2_ trapping is the S_2_ dynamics up to the S_2_/S_1_ crossing. In this region of the potential energy surface, we do not expect any relevant impact of non-dynamic electron correlation, but we are sure that dynamic electron correlation plays a role; for instance, correcting the strong overestimation of the ππ* energy typical of CASSCF predictions [15,19]. Therefore, we have approached the problem through surface hopping simulations based on algebraic diagrammatic construction to second order (ADC(2)) method, which, quite opposite to CASSCF, effectively recovers dynamic correlation but neglects non-dynamic correlation. We can already anticipate that this methodological change had a major impact on the results: the S_2_ trapping is strongly reduced.

## 2. Results

### 2.1. Topography of Excited States

**Figure 1:**
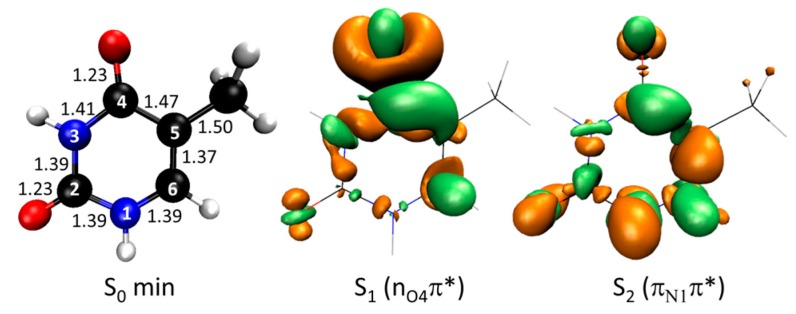
(**Left**) Geometry of ground state thymine with atom numbering and main bond lengths in Å; (**Center**) Difference between the electronic densities of the S_1_ state (nπ*) and of the ground state; (**Right**) Difference between the electronic densities of the S_2_ state (ππ*) and of the ground state. In this figure and throughout the paper, orange surfaces in the density difference indicate electron deficient regions, while green surfaces indicate electron rich regions.

Thymine’s vertical excitation at ADC(2)/(aug-)cc-pVDZ level is characterized by a dark S_1_ state at 4.56 eV with nπ* character and a bright S_2_ excitation at 5.06 eV with a ππ* character (Table 2). Electronic density differences for these two states in comparison to the ground state density are shown in Figure 1.

**Figure 2:**
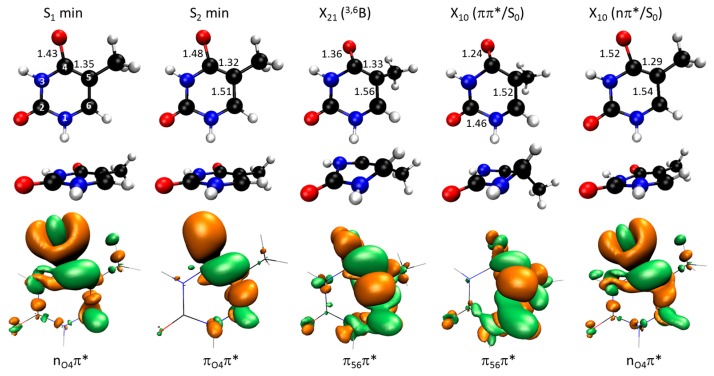
Geometries of the S_1_ and S_2_ minima, and of the X_21_, X_10_ (ππ*/S_0_) and X_10_ (nπ*) intersection points. The bond distances with the largest variation in comparison to the ground state geometry are given in Å. The electronic density difference between the relevant state in each case and the ground state are shown at the bottom.

The main topographic points in these two excited states are the minima on S_2_ and S_1_, the intersection point between S_2_ and S_1_, and the two intersection points between S_1_ and S_0_. They are characterized in Figure 2. Like in the Franck-Condon (FC) region, the S_2_ state around the S_2_ minimum has a ππ* character. Nevertheless, while in the FC region the electron is promoted from a π bond involving N1, C5, and C6, in the S_2_ minimum the electron is promoted from the C4O π bond (compare the electronic density differences in Figure 1 and Figure 2). As a consequence of losing the C4O π bond in the S_2_ minimum, there is a strong stretching of the C4O distance from 1.23 Å in the FC region to 1.48 Å in the S_2_ minimum. We will later discuss how this feature has a major impact on the S_2_ → S_1_ dynamics. Another feature of this minimum is a shrinking of the C4C5 and C5C6 bonds, indicating the formation of π bonds in that region.

The S_1_ state in the S_1_ minimum still has the same nπ* character as in the FC region (electron excitation from C4O). Compared to the ground state geometry, the main geometric consequence of the relaxation into this minimum is the stretching of the C4O bond and the shrinking of the C4C5.

The crossing between S_2_ and S_1_ is reached by an out-of-plane deformation of the ring (Figure 2). At the minimum energy crossing point, the ring assumes a boat conformation with N3 and C6 above the plane (^3,6^B). Along the S_2_ state, this crossing still occurs on a ππ* state, but there is a significant density change in comparison to that of the S_2_ minimum. While in the S_2_ minimum the C4O π bond is lost, in the X_21_ crossing this bond it is recovered. This is clear from the shrinking of the C4O distance from 1.48 to 1.36 Å between these two geometries. In fact, it is exactly this bond formation responsible for the energy stabilization, which ultimately leads to the intersection.

The character change of the ππ* state between the FC region and the S_2_ minimum was first pointed out in ref. [21], while the character change between the S_2_ minimum and the X_21_ intersection was first noticed in ref. [14]. Both works, however, were limited to an analysis of the main molecular orbitals involved in the transitions. The density difference analysis goes a step further, revealing more precisely where the excitations originated.

There are two main minimum energy crossings between S_1_ and S_0_. The first one connects the ππ* state to the ground state (X_10_ π_56_π*/S_0_ in Figure 2). It occurs along the same type of geometrical distortion that gives rise to X_21_. The X_10_ ππ*/S_0_ crossing also features a ^3,6^B boat conformation, but while the puckering degree is Q = 0.48 Å for X_21_, it increases further to Q = 0.54 Å for X_10_ (Q is the Cremer-Pople parameter measuring the degree of puckering in a 6-membered ring [26]). At the crossing, the C4O π bond is fully formed and the C4O distance is 1.24 Å, essentially the same as in the ground state, 1.23 Å.

The second X_10_ crossing connects the nπ* state to the ground state (X_10_ n_O4_π*/S_0_ in Figure 2). It occurs as a further semi-planar distortion of the S_1_ minimum, with the C4O bond stretched to 1.52 Å and the C4C5 bond shrank to 1.29 Å.

**Figure 3:**
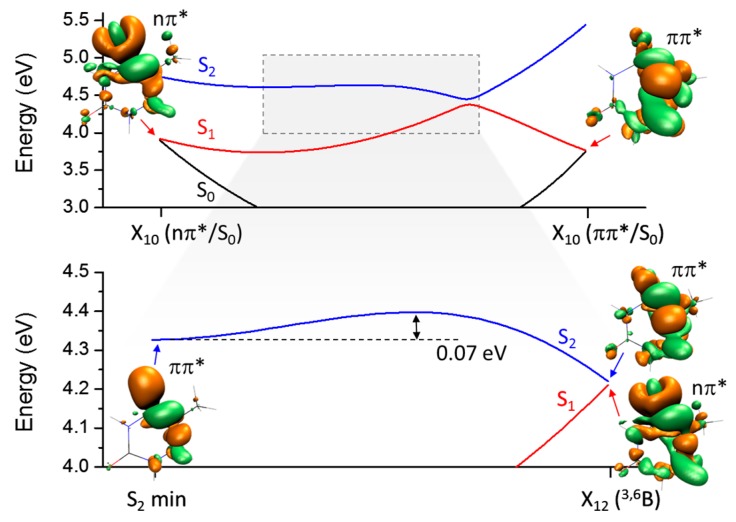
(**Top**) LIIC profile between the two X_10_ intersection points; (**Bottom**) LIIC profile between the S_2_ minimum and the X_21_ intersection point. Electronic density differences at key points are shown as well.

This general topography of the lowest singlet excited states is illustrated in Figure 3. The top graph is the potential energy profile of the S_0_, S_1_, and S_2_ states obtained by linear interpolation of internal coordinates (LIIC) between the two X_10_ intersection points. The bottom graph shows S_1_ and S_2_ along the interpolation between the S_2_ minimum and the X_21_ intersection.

As already mentioned, starting from the S_2_ minimum, X_12_ is reached by an out-of-plane distortion that recovers the C4O bond. With ADC(2), the cost for this bond formation is minimum, only 0.07 eV. For comparison, at CASSCF, the same interpolated barrier is 0.35 eV [14]. Note that these are linearly interpolated values, which overestimate the true barriers. Full optimization of transition states resulted in barriers of 0.25 eV with CASSCF [19] and between 0.01 and 0.05 eV with multi-state complete active space perturbation theory to second order (MS-CASPT2) [12,19].

Although the qualitative description of the excited state topography of thymine obtained with ADC(2) is in agreement with previous description using other computational methods [1,15,19], it is clear from Table 2 that this agreement is merely qualitative. The quantitative description of the minima and intersection energies bears important differences between the methods. Unfortunately, at this point we cannot take for granted even that CASPT2 result would be the most accurate, as the usual protocol of computing CASPT2 energies on CASSCF optimized geometries may result in poor excitation energies, especially near the crossing seam (see, for instance, in Table 2, the large energy splits when MS-CASPT2 is used on CASSCF optimized intersection geometries). With this methodological warning in mind, we will present the dynamics results in the next section and later discuss possible sources of inaccuracy on the ADC(2) surfaces.

### 2.2. Dynamics

**Figure 4:**
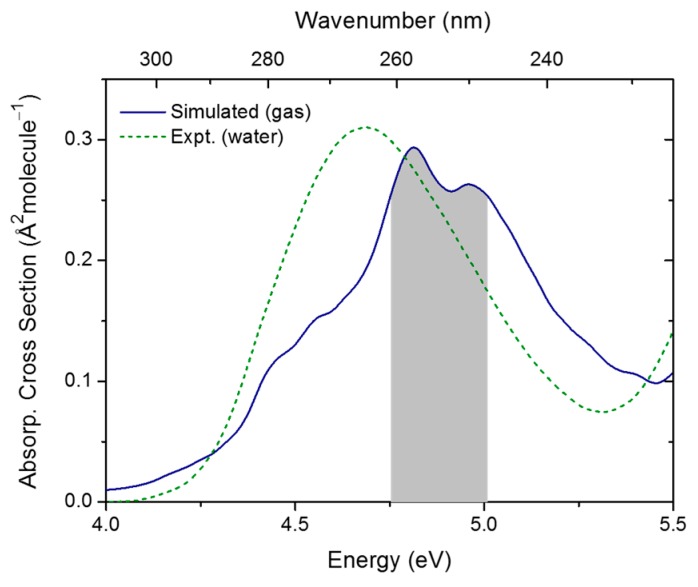
Simulated spectrum of thymine in the gas phase. The shaded area indicates where initial conditions for dynamics where selected from. The dashed line is the experimental spectrum of thymine in water from ref. [27].

Initial conditions for dynamics were obtained by first simulating the absorption spectrum of thymine in the gas phase. This spectrum is shown in Figure 4 compared to the experimental result in water from ref. [27]. The ADC(2)/(aug)-cc-pVDZ absorption band is peaked at 4.89 eV. The experimental gas phase result obtained by electron impact is 4.95 ± 0.08 eV [28]. The absorption intensity and band shape are also in very good agreement with the experimental results in water [27].

**Figure 5:**
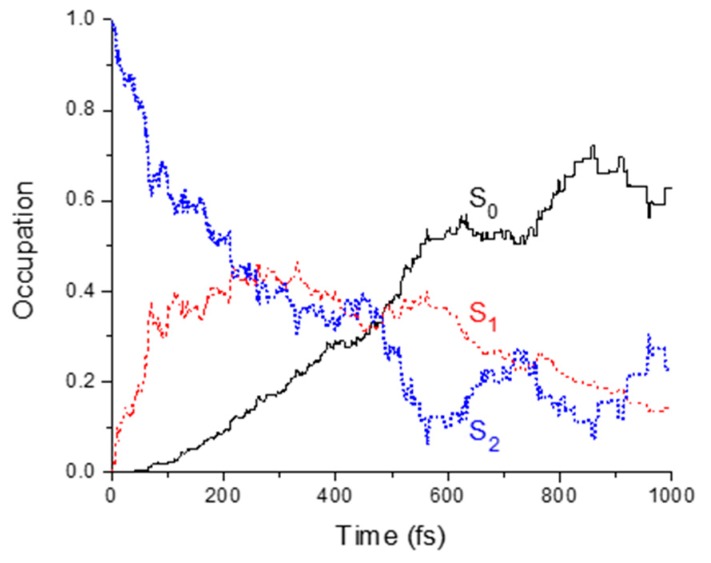
State occupations during dynamics.

ADC(2)/(aug-)cc-pVDZ surface hopping dynamics of thymine in the gas phase shows a fast relaxation process, with S_2_ converting to S_1_, and then S_1_ converting to S_0_ (Figure 5). The fitting of the state occupation (fraction of trajectories in each state) as a function of time shows an S_2_ → S_1_ exponential decay of 84% of the population within 253 fs (Table 3). The fitting of the S_1_ occupation (see Supplementary Materials) reveals that 70% of the population returns to the ground state with a 391 fs time constant. 30% of the total population deactivates with a time constant above 1 ps. Note that considering a confidence level of 90%, our 115 trajectories only allow these fractions to be determined within a maximum statistical uncertainty of ±8%.

**Figure 6:**
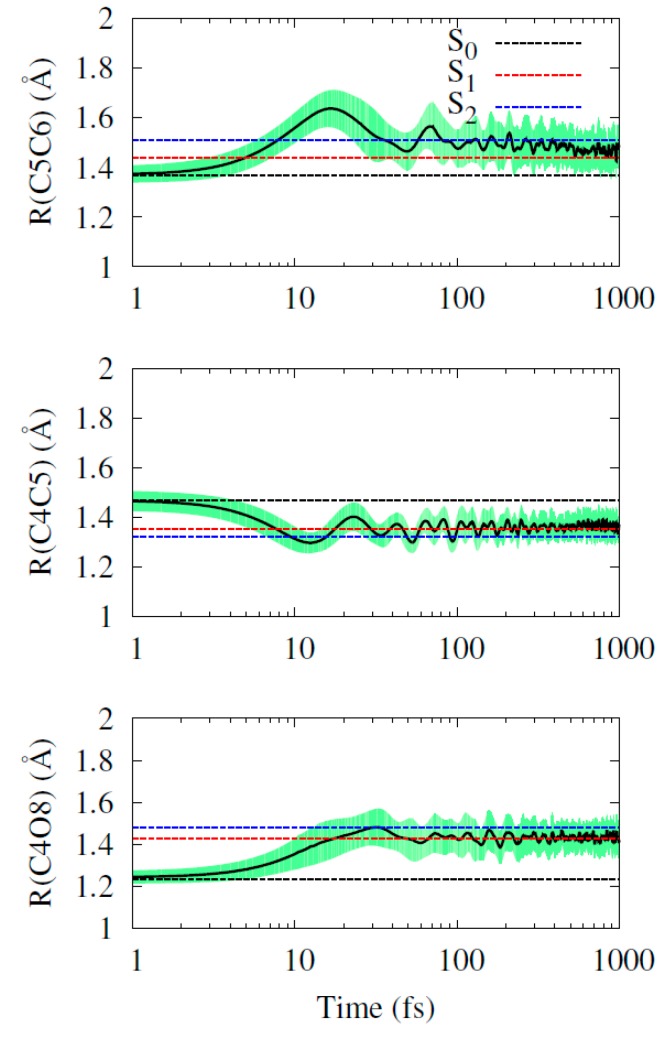
Time evolution of the C5C6 (**Top**), C4C5 (**Middle**), and C4O (**Bottom**) bond distances averaged over all trajectories. The shaded areas show plus-minus one standard deviation around the mean value. Horizontal lines indicate the optimal values of the S_0_, S_1_ and S_2_ minima.

As we discussed in the previous section, the C5C6, C4C5, and C4O bond distances are markedly distinct in the three state minima. Therefore, their evolution during the dynamics is useful to gather further information on the state population. The time evolution of these bond distances averaged over all trajectories are shown in Figure 6. All three start near the optimal S_0_ minimum value. The S_2_ minimum is quickly reached, after 100 fs. This can be clearly seen only in the C5C6 bond, which bears the largest difference between S_1_ and S_2_ minima. In the other two cases, the large number of trajectories quickly decaying to S_1_ (together with the large standard deviation) tends to hide this feature. By the end of the simulations, the three bond distances oscillate near the S_1_ minimum. (As we discuss in the Theoretical and Computational Details, we do not simulate the ground state dynamics. For this reason, in the long term, we do not see the ground state bond distances being recovered.)

**Figure 7:**
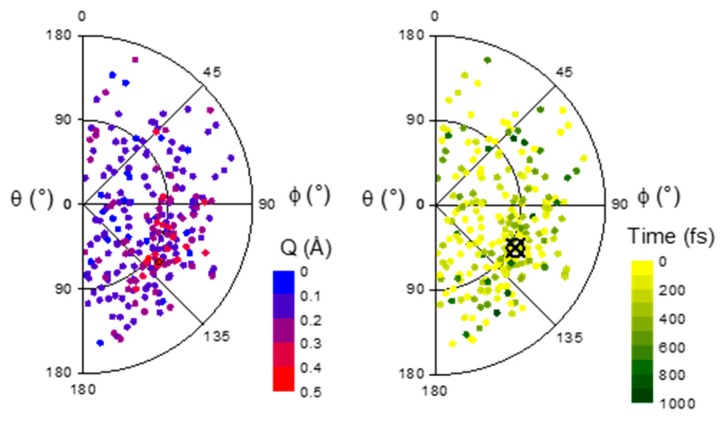
Polar plot showing the distribution of Cremer-Pople parameters θ and φ at the S_2_/S_1_ hop geometry. On the left, the colors additionally indicate the value of the third parameter Q. On the right, the color code indicates the hop time. Both maps were symmetry-projected to show only the φ < 180° region. The crossed circle indicates the minimum energy crossing point.

The S_2_ → S_1_ conversion occurs in a wide variety of ring puckering conformations, including distortions far away from the minimum intersection point. This is illustrated in Figure 7, which shows the distribution of Cremer-Pople parameters θ and φ at the S_2_/S_1_ hop point. (These two parameters characterize the type of puckering in a 6-membered ring.) Larger ring distortions (large Q) tend to occur near the ^3,6^B region (θ = 90°, φ = 120°). There is no correlation between the type of ring puckering and the hop time.

The S_1_ → S_0_ conversion occurs at both branches of intersection, the nπ*/S_0_ and the ππ*/S_0_. From the 84% of the population converting to S_1_, 61% deactivates in the nπ*/S_0_ crossing and 9% in the ππ*/S_0_. Finally, 14% of the population does not decay in the sub-picosecond process and remains in S_1_.

## 3. Discussion

**Figure 8:**
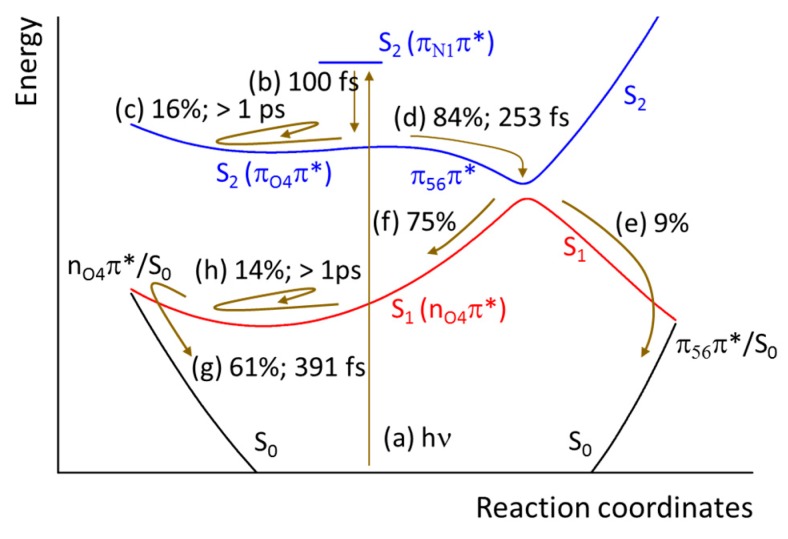
Schematic view of thymine dynamics as predicted by ADC(2) surface hopping. See text for description.

The results of the ADC(2) surface hopping dynamics of thymine in the gas phase are schematically summarized in Figure 8. After photoexcitation into the π_N1_π* state (a), thymine relaxes within 100 fs to the minimum of the S_2_ surface holding a π_O4_π* character (b). A minor fraction of the population is trapped in S_2_ (c), while the remaining flows to S_1_ in about 250 fs (d). This conversion to S_1_ splits the population once more: a minor part follows the S_1_ state along the π_56_π* branch and immediately converts to the ground state (e); the major part, however, flows to the S_1_ n_O4_π* minimum (f). After about 400 fs, most of the population converts to the ground state in the n_O4_π*/S_0_ crossing (g), while a minor fraction remains trapped in the S_1_ state (h).

These results imply that, upon inclusion of dynamic electron correlation in the dynamics, the S_2_ trapping is drastically reduced and may affect only 16% of the population. In CASSCF dynamics, it affects about 80% of the population [14]. This difference is a strong indication that dynamics based on CASSCF [14,23] may have overestimated the role of the S_2_ trapping. The reason for this overestimation is clear: in CASSCF the formation of C4O π bond (which allows to reach the S_2_/S_1_ intersection) has an energetic cost, in the form of a barrier (0.25 eV [19]) separating the S_2_ minimum and the intersection. This barrier practically disappears when dynamic electron correlation is included, either in ADC(2) or in CASPT2.

ADC(2) is a single reference method, whose current implementation is based on linear response theory. Naturally, we cannot expect that it will provide definitive answers on thymine time constants. Moreover, we should consider that we cannot accurately compute the time constant for deactivation to S_0_ due to the lack of S_1_/S_0_ nonadiabatic couplings. As explained later in the section Theoretical and Experimental Details, we deal with this problem using an energy threshold as hop criterion. For this reason, both the S_1_ → S_0_ time constant and fraction of population bear large uncertainties. For instance, if we double the energy gap threshold from 0.15 to 0.30 eV, the S_1_ → S_0_ time constant is reduced from 391 to 291 fs.

In particular, the efficient S_1_/S_0_ conversion of 70% of the population in the sub-picosecond scale is especially challenging to rationalize in view of the experimental signal in the few picoseconds range (Table 1). Even if the third of the population which is left in the excited states decayed with a time constant spanning a few picoseconds, this fraction may be too small to account for the strong ion signal originating from this spectral region. Nevertheless, without a full spectral simulation including the probe process, we also cannot discard the possibility that this third of the population is in fact ultimately responsible for the signal. Unfortunately, the experimental references do not disclose the fitting amplitudes in addition to the time constants. They would be invaluable to check this point.

If the fraction of the population decaying in the picosecond scale is significantly larger than 30%, this will indicate that the nπ*/S_0_ intersection predicted by ADC(2) is too low in energy, which could be result of the wrong topography of the S_1_/S_0_ crossing seam at this level [29]. However, even if we conclude that ADC(2) dynamics is artificially fast, it seems improbable that its prediction of sub-picosecond S_1_/S_0_ conversion is completely wrong. The occurrence of this fast process in thymine should be seriously considered, as it has recurrently shown up in the simulations: it is relevant in ADC(2) dynamics, dominant in semi-empirical OM2/MRCI dynamics [20], and even in CASSCF dynamics it affects about 20% of the population [14]. In practical terms, this means that the current trend of fitting time-resolved spectra of thymine with three exponential decays with fs, ps, and ns time constants may be too strict. We may even recall alternative fittings, like that in ref. [3], which split the sub-picosecond time constant in two, <50 fs and 490 fs.

The photodynamics of thymine has daring experimentalists and theoreticians. Although we are still not in a position to deliver a final assignment of its many spectral features, it is becoming obvious that assigning its time constants to single processes may be the wrong strategy. The ensemble of results points to a situation where several processes contribute to the dynamics in the same time scale. In particular, it is astonishing that in the sub-picosecond time scale alone, the time-resolved spectra may be influenced by laser field, variation of the IP along S_2_ relaxation through three different ππ* characters, S_2_/S_1_ conversion, and S_1_/S_0_ conversion in two different branches of the crossing seem.

To learn how to resolve each of them is the next challenge.

## 4. Theoretical and Computational Details

### 4.1. Potential Energy, Spectrum, and Dynamics Simulations

The geometries of the ground and the first two singlet excited states of thymine were optimized with algebraic diagrammatic construction to second order (ADC(2)) level [30,31] (for the ground state, on MP2 level). The Dunning’s aug-cc-pVDZ basis set was used for all elements except for hydrogen, where cc-pVDZ was employed [32]. This mixed basis set is denoted (aug-)cc-pVDZ in the text. Calculations were done with frozen core and applying the resolution-of-identity (RI) approximation for the computation of two-electron integrals. In addition to state minima, we also optimized two intersection minima between S_0_ and S_1_ states (denoted X_10_), and an intersection minimum between S_2_ and S_1_ (denoted as X_21_). Reaction paths were computed applying linear interpolation in natural internal coordinates (LIIC) [33].

We simulated the photoabsorption spectrum of thymine applying the nuclear ensemble approach [34]. A set of 500 molecular geometries and momenta was created using harmonic-oscillator Wigner distribution based on normal modes in the ground state. Vertical excitation energies and oscillator strengths for transitions to the first ten singlet states were computed using ADC(2)/(aug-)cc-pVDZ for each geometry in the ensemble.

We performed nonadiabatic excited-state dynamics simulations using surface hopping on ADC(2)/(aug-)cc-pVDZ potential energy surfaces. The initial conditions (geometries and momenta) for dynamics simulations were selected starting from the bright S_2_ state. They were filtered from the initial ensemble of 500 initial conditions, from within the 4.88 ± 0.13 eV energy window, which includes the maximum of the first band in the spectrum. This procedure produced 115 initial conditions, which were propagated for a maximum 1 ps.

Nonadiabatic events between S_2_ and S_1_ were taken into account by the fewest switches algorithm [35] corrected for decoherence effects (α = 0.1 Hartree) [36]. Because of the limitation of ADC(2) to deal with multi-reference ground states [29], trajectories were stopped whenever their S_1_/S_0_ energy gap dropped below 0.15 eV. The corresponding time step was taken as an estimate of the S_1_/S_0_ crossing time. Newton’s equations of motion were integrated using the velocity Verlet algorithm [37] with the time step of 0.5 fs. Integration of the semi-classical Schrödinger equation was done employing the 5th order Butcher’s algorithm [38] with time step of 0.025 fs, using interpolated electronic properties between the classical steps. Computation of nonadiabatic couplings between excited states is described in the next section.

To analyze the distortions of thymine’s ring during dynamics, we computed the Cremer-Pople parameters [26] and classified them into conformations according to Boeyens’ scheme [39].

All ADC(2) computations were done with TURBOMOLE [40]. Spectrum and dynamics were computed with the NEWTON-X/TURBOMOLE interface [41,42]. Intersection point optimizations were done with an in-house modified version of CIOpt program [43]. Cremer-Pople parameters were obtained using the PLATON program [44].

### 4.2. OD Method for Coupling Calculations

Nonadiabatic couplings σ_*mn*_ between electronic states *m* and *n* can be dynamically estimated on the basis of the time derivative of the corresponding wave functions during the trajectory:

(1) $$\sigma_{mn}=\langle \Psi_{m}|\partial_{t}\Psi_{n}\rangle .$$

When computed by finite differences, time-derivative nonadiabatic couplings (TDNC) σ_*mn*_ can be conveniently written in terms of wave function overlaps between consecutive time steps. Then, as proposed by Hammes-Schiffer and Tully [45], TDNC can be used to evaluate the fewest-switches probability formula, by directly replacing the inner product between the nonadiabatic coupling vector and the nuclear velocities, σ_*mn*_ = **F**_*mn*_**·v**. This procedure has become popular, as it allows us to overcome the cumbersome evaluation of nonadiabatic coupling vectors [46,47,48].

In the present work, TDNC are obtained by evaluating Equation (1) with the OD (for Orbital Derivative) method proposed in ref. [49]. This method requires computation of time derivatives (and wave function overlaps) on a basis of molecular orbitals, rather than on a basis of Slater determinants as usually done. (This latter approach will be referred as the DD (for Determinant Derivative) method).

The OD method is discussed in detail in ref. [49]. Here, we briefly outline the main points to explain its current implementation in NEWTON-X. Considering a configuration interaction expansion of singly excited determinants (CIS) $|\Phi_{i}^{a}\rangle ={\hat{a}}_{a}^{+}{\hat{a}}_{i}|\Phi_{0}\rangle$, the electronic wave function for state *m* is:

(2) $$|\Psi_{m}\rangle =\sum_{ia}C_{ia}^{m}|\Phi_{i}^{a}\rangle .$$

The couplings between the excited states *m* and *n* can be evaluated as

(3) $$\sigma_{mn}=\sum_{ia}C_{ia}^{m}\partial_{t}C_{ia}^{n}+\sum_{iab}C_{ia}^{m}C_{jb}^{n}\langle \varphi_{a}|\partial_{t}\varphi_{b}\rangle -\sum_{ija}P_{ij}C_{ia}^{m}C_{jb}^{n}\langle \varphi_{j}|\partial_{t}\varphi_{i}\rangle ,$$

where $P_{ij}$ is a phase that depends on the ordering convention adopted for the molecular orbitals $\left\{\varphi_{k}\right\}$ in the Slater determinants.

Considering the overlap matrix between molecular orbitals from two consecutive time steps, the time derivatives of the molecular orbitals are evaluated by finite differences:

(4) $$\langle \varphi_{j}|\partial_{t}\varphi_{i}\rangle \approx \frac{\langle \varphi_{j}(t)|\varphi_{i}(t+\Delta t)\rangle }{\Delta t}\equiv \frac{S_{ji}(t,t+\Delta t)}{\Delta t},$$

where $S_{ji}$ is the orbital overlap matrix. An orbital phase matching algorithm is used to assure the continuity of orbitals at different time steps.

The formal scaling of the TDNC evaluation is reduced from $N_{occ}^{5}N_{virt}^{2}$ in the DD approach to $N_{occ}N_{virt}^{2}$ in the OD. This method has had excellent results in comparison to the DD at significantly lower computational costs [49]. In the present simulations of thymine, for instance, computation of TDNC with the OD method was ten times faster than with the DD method.

We have implemented the OD method in NEWTON-X, where it is available for interfaces with GAUSSIAN [50] (CIS, TDA, and TDDFT methods) and TURBOMOLE (TDA, TDDFT, CC2, and ADC(2) methods). In particular, for the density functional based methods, approximated CIS wave functions are built using the Casida ansatz [51,52]. In the case of ADC(2) and CC2, approximated CIS wave functions are expressed in terms of Jacobian eigenvectors, where double excitations are neglected and the resulting wave functions are reorthonormalized [53].

## Figures and Tables

**Table 1:** Excited-state time constants of thymine in the gas phase according to the experiments under various pump and probe conditions.

Pump (nm)	Probe (nm)	τ_1_ (fs)	τ_2_ (ps)	τ_3_ (ps)	τ_4_ (ns)	References
250	200	<50	0.49	6.4		[3]
260	295	175		6.13	>1	[1]
266	2.19 (X-ray)	200–300				[4]
266	400/800	<100		7	long	[5]
266	800	200		7		[6]
267	2 × 400	105		5.12		[7]
267	800	100		7	>1	[8]
267	800			6.4	>100	[2]
270	193				293	[9]
272	800	130		6.5		[10]

**Table 2:** Ground and excited singlet state energies of the minima and intersection points of thymine in the gas phase obtained with ADC(2), CASSCF, and MS-CASPT2. All energies are relative to the ground state minimum.

Geometry	State	Energy (eV)		
		ADC(2)	CASSCF ^a^	MS-CASPT2 ^b^
S_0_ min	S_0_ (cs)	0.00	0.00	0.00
	S_1_ (n_O4_π*)	4.56	5.19	5.09
	S_2_ (π_N1_π*)	5.06	6.87	5.09
S_1_ min	S_0_ (cs)	1.33	1.39	1.02
	S_1_ (n_O4_π*)	3.33	4.02	4.37
S_2_ min	S_0_ (cs)	2.14	1.71	1.28
	S_1_ (n_O4_π*)	3.50	4.18	4.51
	S_2_ (π_O4_π*)	4.18	5.64	4.77
X_10_ (nπ*/S_0_)	S_0_ (cs)	3.90	5.02	5.02
	S_1_ (n_O4_π*)	3.90	5.13	5.60
X_10_ (ππ*/S_0_)	S_0_ (cs)	3.82	4.49	4.19
	S_1_ (π_56_π*)	3.82	5.54	4.41
X_21_ (^3,6^B)	S_0_ (cs)	3.37	2.68	2.23
	S_1_ (n_O4_π*)	4.21	5.61	4.79
	S_2_ (π_56_π*)	4.22	6.00	5.63

^a^ CASSCF(12,9)/6-311G* and ^b^ MS-CASPT2(12,9)/6-311G* on CASSCF(8,6)/6-31G* geometries; data from ref. [19].

**Table 3:** Time constants for different processes and corresponding fractions of population being affected by them. For the S_2_ → S_1_ and S_1_ → S_0_ processes, parameters were obtained by fitting the state occupations in Figure 5 with the kinetic model discussed in the Supplementary Material. For FC → S_2_ min, the information was extracted from Figure 6.

Process	*f*_τ_	τ (fs)
FC → S_2_ min	1.00	~100
S_2_ → S_1_	0.84	253
S_1_ → S_0_	0.70	391

## Supplementary Materials

The following are available online at: http://www.mdpi.com/1420-3049/21/11/1603/s1, kinetic model to fit occupations and Cartesian coordinates for all structures.
