# Supplementary material for: New Insights into the State Trapping of UV-Excited Thymine
Source: Molecules. 2016 Nov 23;21(11):1603. doi: 10.3390/molecules21111603 (PMC6273395; doi:10.3390/molecules21111603)
Supplement: Supplementary file 1 [file molecules-21-01603-s001.pdf]

Supplementary Materials: New Insights into the State Trapping of UV-Excited Thymine

Ljiljana Stojanović, Shuming Bai, Jayashree Nagesh, Artur F. Izmaylov, Rachel Crespo-Otero, Hans Lischka and Mario Barbatti

1. Kinetic Model for Occupation Fitting

Consider the set of reactions in the scheme below.

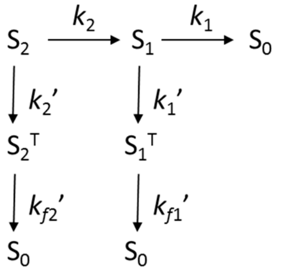

Part of S_2 population relaxes to S_1 with rate k_2, while another part is trapped in S_2^T until it later relaxes to S_0 with k_{f2}'. By construction, $k_{f2}' << k_2$ and k_2'. The same is valid for S_1, with rates $k_1 << k_1'$ and k_{f1}'. Rewriting $k_2' = (\alpha - 1) k_2$ and $k_1' = (\beta - 1) k_1$, the kinetic equations for this set of reactions have as solution the equations (S1).

$$
\begin{aligned}
S_2(t) &= S_{20} \exp(-\alpha k_2 t) \\
S_2^T(t) &= \frac{(\alpha - 1)}{\alpha} S_{20} \left[1 - \exp(-\alpha k_2 t)\right] \\
S_1(t) &= \frac{k_2 S_{20}}{\beta k_1 - \alpha k_2} \left[\exp(-\alpha k_2 t) - \exp(-\beta k_1 t)\right] \\
S_1^T(t) &= \frac{k_1 (\beta - 1) k_2 S_{20}}{\beta k_1 - \alpha k_2} \left[\frac{1 - \exp(-\alpha k_2 t)}{\alpha k_2} - \frac{1 - \exp(-\beta k_1 t)}{\beta k_1}\right] \\
S_0(t) &= \frac{k_2 k_1}{\beta k_1 - \alpha k_2} \left[\frac{1 - \exp(-\alpha k_2 t)}{\alpha k_2} - \frac{1 - \exp(-\beta k_1 t)}{\beta k_1}\right]
\end{aligned}
\quad \text{(S1)}
$$

Asymptotically, the population trapped in S_2 is $(1 - 1/\alpha)$ and in S_1 is $(\beta - 1)/\alpha\beta$ ($S_{20} = 1$). The time constant for the fast decay from S_2 to S_1 is $1/k_2$. The time constant for the fast decay from S_1 to S_0 is $1/k_1$.

Asymptotic populations and time constants were obtained by fitting the S_2 state occupation with the function $S_2(t) + S_2^T(t)$ and the S_1 state occupation with $S_1(t) + S_1^T(t)$.

2. Cartesian coordinates

Cartesian coordinates of minima and intersection points optimized with ADC(2)/(aug-)cc-pVDZ (in Å).

S0 min

N	−3.757921	0.391909	−0.457421
C	−2.955367	−0.721660	−0.672991
N	−1.599944	−0.434009	−0.563253
C	−0.990970	0.802030	−0.271308
C	−1.928450	1.912081	−0.061501
C	−3.265663	1.653150	−0.164921

O	−3.401520	−1.837122	−0.932248
O	0.237798	0.893292	−0.208360
C	−1.365380	3.269833	0.255801
H	−4.756137	0.224830	−0.528884
H	−0.971100	−1.221558	−0.713817
H	−4.022615	2.429164	−0.021755
H	−2.172402	4.009748	0.370491
H	−0.685924	3.602328	−0.544247
H	−0.776189	3.236749	1.185509

S1 min

N	−3.749358	0.340197	−0.303378
C	−2.950857	−0.721762	−0.667756
N	−1.581449	−0.423846	−0.637897
C	−1.112995	0.866668	−0.382220
C	−1.901943	1.913089	−0.050927
C	−3.312746	1.636664	0.006639
O	−3.370156	−1.838220	−0.980590
O	0.311020	0.871753	−0.502860
C	−1.351272	3.284322	0.251723
H	−4.741649	0.122680	−0.306485
H	−0.963579	−1.140341	−1.010360
H	−4.074116	2.374324	0.263236
H	−1.749343	4.035453	−0.448920
H	−0.251840	3.291133	0.165842
H	−1.611502	3.598649	1.275047

S2 min

N	−3.746974	0.367199	−0.306531
C	−2.952425	−0.732122	−0.655535
N	−1.603630	−0.405055	−0.697130
C	−1.111272	0.867492	−0.411555
C	−1.850471	1.901685	−0.049262
C	−3.335441	1.630815	0.037271
O	−3.417754	−1.845691	−0.899421
O	0.361895	0.829995	−0.548825
C	−1.343547	3.275823	0.279002
H	−4.741293	0.139764	−0.297041
H	−0.931388	−1.125187	−0.954763
H	−4.092843	2.418849	0.083351
H	−1.788478	4.029300	−0.393251
H	−0.249054	3.306305	0.171019
H	−1.609102	3.551588	1.313768

X10 pp*/S0

N	−6.472869	0.415164	−0.054546
C	−7.676460	1.083781	0.424943
N	−7.515171	2.450152	0.601444

C	−6.660789	3.235047	−0.284851
C	−5.535512	2.494450	−0.730766
C	−5.342296	1.150553	−0.051520
O	−8.663297	0.427524	0.721114
O	−7.082789	4.331444	−0.690862
C	−5.226347	2.504643	−2.212824
H	−6.477878	−0.580395	0.185573
H	−8.394352	2.921515	0.823404
H	−4.449496	0.803497	0.483144
H	−4.155421	2.696060	−2.406538
H	−5.818679	3.299262	−2.693194
H	−5.478564	1.530834	−2.677541

X10 np*/S0

N	−3.734396	0.337524	−0.272188
C	−2.934778	−0.716795	−0.640752
N	−1.541435	−0.488071	−0.498846
C	−1.176345	0.867257	−0.322312
C	−1.865359	1.901915	0.006483
C	−3.355496	1.598381	0.241963
O	−3.358960	−1.803751	−1.044581
O	0.324714	0.800016	−0.525806
C	−1.342894	3.289332	0.250024
H	−4.722158	0.094920	−0.310527
H	−1.021322	−1.042711	−1.180291
H	−4.071546	2.405623	0.023747
H	−1.776499	4.001943	−0.470916
H	−0.243661	3.332008	0.146860
H	−1.591655	3.633161	1.268223

X21 pp*/np*

N	−3.696326	0.430982	0.030353
C	−2.857771	−0.686754	−0.309832
N	−1.660106	−0.300695	−0.893176
C	−1.051456	0.938355	−0.481788
C	−1.834791	1.955446	−0.132858
C	−3.355149	1.691555	−0.336062
O	−3.252097	−1.837558	−0.158375
O	0.290048	0.826474	−0.304954
C	−1.435356	3.082637	0.776127
H	−4.670513	0.147567	0.168662
H	−1.007186	−1.072701	−1.023189
H	−4.056672	2.317383	−0.908713
H	−1.601300	4.067821	0.307413
H	−0.369466	2.982077	1.044713
H	−2.038288	3.057581	1.703737
